# Comparison of Fruits of *Forsythia suspensa* at Two Different Maturation Stages by NMR-Based Metabolomics

**DOI:** 10.3390/molecules200610065

**Published:** 2015-05-29

**Authors:** Jinping Jia, Fusheng Zhang, Zhenyu Li, Xuemei Qin, Liwei Zhang

**Affiliations:** 1 Institute of Molecular Science, Shanxi University, No.92, Wucheng Road, Taiyuan 030006, Shanxi, China; E-Mail: jiajp@sxu.edu.cn; 2 Modern Research Center for Traditional Chinese Medicine, Shanxi University, No.92, Wucheng Road, Taiyuan 030006, Shanxi, China; E-Mails: ample1007@163.com (F.Z.); lizhenyu@sxu.edu.cn (Z.L.); qinxm@sxu.edu.cn (X.Q.)

**Keywords:** NMR, metabolomics, maturation stage, *Forsythia suspensa*

## Abstract

Forsythiae Fructus (FF), the dried fruit of *Forsythia suspensa*, has been widely used as a heat-clearing and detoxifying herbal medicine in China. Green FF (GF) and ripe FF (RF) are fruits of *Forsythia suspensa* at different maturity stages collected about a month apart. FF undergoes a complex series of physical and biochemical changes during fruit ripening. However, the clinical uses of GF and RF have not been distinguished to date. In order to comprehensively compare the chemical compositions of GF and RF, NMR-based metabolomics coupled with HPLC and UV spectrophotometry methods were adopted in this study. Furthermore, the *in vitro* antioxidant and antibacterial activities of 50% methanol extracts of GF and RF were also evaluated. A total of 27 metabolites were identified based on NMR data, and eight of them were found to be different between the GF and RF groups. The GF group contained higher levels of forsythoside A, forsythoside C, cornoside, rutin, phillyrin and gallic acid and lower levels of rengyol and β-glucose compared with the RF group. The antioxidant activity of GF was higher than that of RF, but no significant difference was observed between the antibacterial activities of GF and RF. Given our results showing their distinct chemical compositions, we propose that NMR-based metabolic profiling can be used to discriminate between GF and RF. Differences in the chemical and biological activities of GF and RF, as well as their clinical efficacies in traditional Chinese medicine should be systematically investigated in future studies.

## 1. Introduction

Forsythiae Fructus (FF), the dried fruit of *Forsythia suspensa*, is widely used as an antipyretic, antidotal and anti-inflammatory agent in China, Japan and Korea for treating infections, such as acute nephritis, erysipelas and ulcers [1,2]. It also suppresses vomiting, inhibits elastase activity, resists hepatic injury and exhibits diuretic, analgesic, antioxidant and antiviral effects [3]. In addition to being a sore-effective medicine, FF is also a popular heat-clearing and detoxifying herb; indeed, more than 40 Chinese medicinal preparations containing FF are listed in the Chinese pharmacopoeia [4]. Some components of FF include phenylethanoid glycosides, lignans, flavonoids, terpenes, alkaloids and volatile oils [5]. Among them, phenylethanoid glycosides, lignans and flavonoids, which are phenolic compounds, have been reported to exhibit diverse biological activities [6,7,8,9].

Depending on the maturity stage of *F. suspensa*, two kinds of FF exist—green FF (GF) and ripe FF (RF). GF (called *Qingqiao* in Chinese) is usually harvested in early September, while the fruits are still greenish, but nearly ripe and indehiscent. As such, GF requires thorough steaming and drying under the Sun. RF (called *Laoqiao* in Chinese) is usually harvested in October, and its fruits are fully ripe, yellow, dehiscent and dried. Both GF and RF are listed in the Chinese pharmacopoeia as FF [4].

Previous studies adopted analytical techniques based on high-performance liquid chromatography (HPLC) to compare GF and RF. Their results showed that the contents of some constituents, such as forsythoside A, phillyrin and rutin, were significantly higher in GF than in RF [8,9,10]. GF is used more frequently in traditional Chinese medicine (TCM) prescriptions, owing in part to its high content of some effective constituents. Because of this, GF is often over-harvested, which, in turn, decreases RF supplies. A contradiction also exists in the traditional view that RF was used as FF in ancient China. Thus, studying the differences between GF and RF is both necessary and urgent. To identify differences and similarities between GF and RF, non-targeted chemical fingerprinting that can cover a wide range of metabolites is necessary.

Metabolomics has recently emerged as an important approach for modern research on medicinal plants and represents a systematic approach for both qualitative and quantitative analysis of metabolite changes [11,12]. The major techniques used for plant metabolomics include nuclear magnetic resonance (NMR), liquid chromatograph-mass spectrometry (LC-MS) and gas chromatograph-mass spectrometry (GC-MS). Among these, NMR is regarded as the most suitable and adequate method to carry out metabolomic analyses, because of its simple sample preparation method, simultaneous detection of a diverse range of abundant primary and secondary metabolites, high reproducibility and stability [13,14]. Furthermore, NMR spectra can reveal detailed structural information, including chemical shifts and coupling constants. NMR can be used for relative quantification and permits direct comparison of concentrations of all compounds present in the sample, as the signal intensity is only dependent on the molar concentration of the solutes [12,13]. The ^1^H-NMR-based metabolomics approach has been widely applied to reveal metabolic differences among herbal medicines, such as *Sambucus ebulus* [14], *Crocus sativus* L. [15] and *Rehmanniae Radix* [16]. Some drawbacks of metabolomics based on NMR analysis include low sensitivity and signal overlap in complex mixtures [13,14]. In the present study, NMR-based metabolomics was performed to compare the chemical differences between GF and RF. *In vitro* biological activities, including antioxidant and antibacterial effects, were also assayed. The results may facilitate rediscovery or determination of the differences between GF and RF, which will positively contribute to the protection of FF wild resources and appropriate clinical use of this herb.

## 2. Results

### 2.1. NMR Metabolic Profiling

The samples were extracted by the CHCl_3_–MeOH–H_2_O system and then divided into chloroform and aqueous methanol fractions. Based on preliminary experimental results, more valuable chemical information was obtained from the ^1^H-NMR spectra of aqueous methanol fractions than from those of chloroform fractions. In the ^1^H-NMR spectra of chloroform fractions, the dominant signals originated from glycerol esters, as well as saturated and unsaturated fatty acids; multivariate data analysis showed no obvious difference between GF and RF (Figures S1 and S2). Thus, the ^1^H-NMR spectra of aqueous methanol fractions were further studied. ^1^H-NMR spectra signals were assigned based on comparisons with the chemical shift of authentic standards, metabolites in the Biological Magnetic Resonance Data Bank [17] and NMR data in the literature (Table 1).

**Table 1:** Relative levels of metabolites detected by NMR in green Forsythiae Fructus (FF) (GF) and ripe FF (RF) (mean ± SD, *n* = 10). VIP, variable influence on projection.

No.		Chemical Shift (δ, ppm), Assignments and Coupling Constants (*J*, Hz)	Identification Compound			GF/RF (Fold Change)		VIP Value		Reference
1		δ 7.63 (H-7′, d, 16.2), 7.16 (H-2′, d, 2.0), 7.06 (H-6′, dd, 1.8, 8.4), 6.90 (H-5′, d, 8.4), 6.81 (H-5, d, 8.4), δ 6.80 (H-2, brs), 6.68 (H-6, dd, 1.8, 7.8), 6.37 (H-8′, d, 16.2), 4.66 (H-1, d, 1.2), 4.46 (H-1’’, d, 7.8), 2.84 (H-7, m), 1.22 (H-6′′′, d, 6.0)	Forsythoside A ^a^			5.07↓ ***		2.65		[18,19]
2		δ 7.65 (H-7′,d, 15.6), 7.16 (H-2′,d, 1.8), 6.80 (H-5, d, 8.4), 6.68 (H-6, dd, 1.8, 7.8), 6.41 (H-8′,d, 16.2), 4.53 (H-1, d, 8.4), 1.29 (H-6′′′, d, 6.6)	Forsythoside C ^a^			2.78↓ ***		1.40		[1,20]
3		δ 7.69 (H-2′, d, 1.8), 6.31 (H-6, d, 2.0), 6.53 (H-8, d, 2.0), 5.01 (H-1′′, d, 7.7), 4.52 (H-1′′′, d, 2.0), 1.12 (H-6′′′, d, 6.1)	Rutin ^a^			2.62↓ ***		1.18		[21]
4		δ 7.53 (H-3, brs), 5.16 (H-1, d, 6.0),	Adoxosidic acid ^a^			0.83↑		0.46		[22]
5		δ 7.18 (H-2′, d, 8.4), 7.08 (H-2, d, 1.8), 7.0 (m), 4.57 (H-7, d, 6.6), 4.16 (d, 9.6)	Phillyrin ^a^			1.35↓ ***		1.66		[23]
6		δ 3.22 (N (CH_3_), s)	Choline ^c^			1.05↓		0.20		[21]
7		δ 7.11 (H-2, H-6, d, 10.2), 6.24 (H-3, H-5, dd, 1.8, 10.2), 4.33 (H-8, d, 7.8), 2.13 (H-7, t, 6.6)	Cornoside ^c^			3.87↓ ***		2.16		[24,25]
8		δ 3.75 (H-8, t, 7.2), δ1.73 (H-7, t, 7.8), δ 1.60 (H-3, H-5, m), δ 1.46 (H-2, H-6, m)	Rengyol ^c^			0.10↑ ***		5.27		[26,27]
9		δ 7.00 (H-2, H-6, s)	Gallic acid ^a^			1.87↓ ***		1.73		[21]
10	δ 2.56 (m), 1.25 (γ-CH_3_, d, 6.6)			3-Hydroxybutyric acid ^c^	0.62↑ ***		0.47		[28]		
11	δ 1.94 (CH_3_, s)			Acetic acid ^c^	0.83↑		0.70		[16,21]		
12	δ 2.35 (CH_3,_ s)			Pyruvic acid ^c^	0.71↑		0.38		[28]		
13	δ 2.46 (CH_2,_ s)			Succinic acid ^c^	1.19↓		0.67		[16,21]		
14	δ 4.30 (α-CH, dd, 9.6, 3.6), 2. 71 (β-CH, dd, 15.6, 3.6), 2.30 (β′-CH dd, 15.0, 9.6)			Malic acid ^c^	0.69↑		0.80		[16,21]		
15	δ 6.55 (α-CH, s)			Fumaric acid ^c^	0.63↑ *		0.22		[21]		
16	δ 8.48(H-COOH, s)			Formic acid ^c^	0.23↑ **		0.06		[21]		
17	δ 4.55(H-1, d, 9)			β-Xylose ^c^	0.91↑		0.64		[16]		
18	δ 4.60(H-1, d, 7.8)			β-Glucose ^b,c^	0.61↑ ***		1.15		[16,21]		
19	δ 5.20(H-1, d, 3.8)			α-Glucose ^b,c^	0.98↑		0.46		[16,21]		
20	δ 4.97 (H-1, d, 3.6Hz), 5.43 (H-1, d, 4.2 Hz)			Raffinose ^c^	0.89↑ **		0.68		[16]		
21	δ 5.42 (Glu-H-1, d, 3.6), 4.19 (Fru-H-1, d, 8.4)			Sucrose ^b,c^	3.33↓ **		0.97		[21]		
22	δ 1.03 (γ-CH_3_, d,7.2 Hz), 0.96 (β-CH_3_, t, 7.2 Hz)			Isoleucine ^b,c^	0.74↑ **		0.66		[16,28]		
23	δ 0.98 (δ-CH_3_, t, 6 Hz)			Leucine ^b,c^	0.56↑ **		0.59		[16,28]		
24	δ 2.27 (m), 1.06 (γ′-CH_3_, d, 7.2), 1.02 (γ-CH_3,_ d, 7.2)			Valine ^b,c^	0.75↑		0.26		[21]		
25	δ 1.33 (CH_3_, d, 6.6)			Threonine ^b,c^	0.59↑ **		0.80		[21]		
26	δ 1.50 (α-CH, d, 7.2)			Alanine ^b,c^	0.28↑ ***		0.73		[16,28]		
27	δ 7.40 (Ar-CH, m), 7.31 (Ar-CH, d, 6.6)			Phenylalanine ^b,c^	1.90↓ *		0.38		[21]		

Values indicate fold change in peak area as % values of the GF group compared with the RF group. ^a^ Metabolites identified by comparison with authentic standards; ^b^ metabolites identified by comparison with the Biological Magnetic Resonance Data Bank; ^c^ metabolites identified by comparison with the literature; * RF group compared with the GF group, *p* < 0.05; ** RF group compared with the GF group, *p* < 0.01; *** RF group compared with the GF group, *p* < 0.001. ↑ Higher in GF; ↓ Lower in GF.

**Figure 1:**
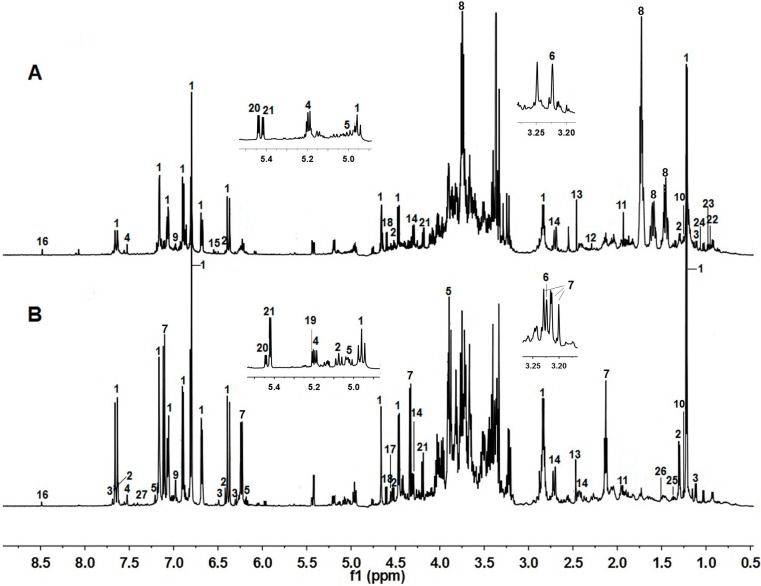
Typical ^1^H-NMR spectra (600 MHz) from aqueous methanol fractions of RF (**A**) and GF (**B**). The numbers correspond to the metabolites in Table 1.

Representative ^1^H-NMR spectra of aqueous methanol fractions of GF and RF are shown in Figure 1, with metabolites indicated based on their chemical shifts and coupling constants. A total of 27 metabolites were identified (Table 1). These metabolites consisted of amino acids, including leucine, isoleucine, valine, threonine, alanine and phenylalanine; organic acids, including 3-hydroxybutyric acid, acetic acid, pyruvic acid, succinic acid, malic acid and formic acid; sugars, including sucrose, β-glucose, α-glucose, β-xylose and raffinose; and other compounds, such as choline, gallic acid, forsythoside A, forsythoside C, cornoside, rengyol, phillyrin, adoxosidic acid and rutin (Figure S3). The characteristic signals of phenylethanoid glycosides were also prominent in the ^1^H-NMR spectra. For example, one of the major compounds was identified as forsythoside A, with resonances at δ 6.80 (H-2, brs), 6.81 (H-5, d, *J* = 8.4), 6.68 (H-6, dd, *J* = 1.8, 7.8) for the 3,4-dihydroxyphenylethyl moiety; and δ 7.16 (H-2′, d, *J* = 2.0), 7.06 (H-6′, dd, *J* = 1.8, 8.4), 6.90 (H-5ʹ, d, *J* = 8.4), as well as two trans-olefinic protons δ 6.37 (H-7′ d, *J* = 16.2) and 7.63 (H-8′, d, *J* = 16.2) for the caffeoyl moiety, together with δ 4.46 (H-1′′, d, *J* = 8.4) for β-glucose, δ 4.66 (H-1′′′, d, *J* = 1.2) and 1.22 (H-6′′′, d, *J* = 6.0) for α-rhamnose. These assignments were further confirmed by comparing with authentic standards.

**Figure 2:**
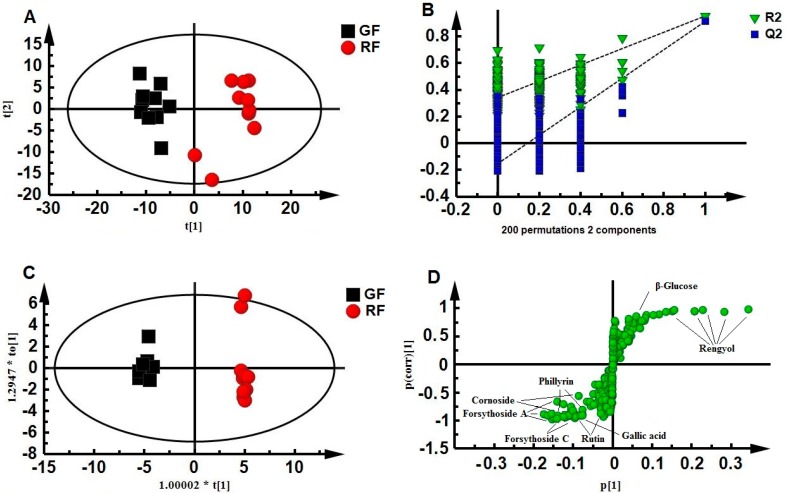
PCA score plots (**A**), permutation test with 200 permutations of the partial least square discriminant analysis (PLS-DA) model, R^2^ = (0.0, 0.353), Q^2^ = (0.0, −0.111); (**B**) orthogonal PLS-DA (OPLS-DA) score plots (**C**) and S-plot (**D**) obtained from NMR metabolic profiles derived from aqueous methanol fractions of GF and RF: GF, black square; RF, red dot.

Based on the assignments of ^1^H-NMR spectra, chemical classification of all samples was performed by multivariate data analysis, which aimed to highlight the differences between GF and RF. An unsupervised approach consisting of principal component analysis (PCA), a nonparametric method of classification, was used to reduce the dimensions of multivariate problems. PCA score plots showed that GF and RF were clearly clustered into two groups, with the first two components explaining 55.0% of total variance (Figure 2A). This finding indicated that GF and RF were significantly different in their metabolites. However, the two samples diverged from other RF samples possibly because of different harvest times. The partial least square discriminant analysis (PLS-DA) model was also validated by a permutation test with 200 permutations (Figure 2B). R^2^ = (0.0, 0.353) and Q^2^ = (0.0, −0.111) indicated that the PLS-DA pattern combined with the loading plot could determine potential biomarkers. To further identify the significant metabolites contributing to distinction between GF and RF, orthogonal partial least square discriminate analysis (OPLS-DA) of these NMR data was further performed. The OPLS-DA score plots demonstrated a clear separation between the GF and RF groups (R^2^X = 0.863, R^2^Y = 0.995, Q^2^ = 0.900) (Figure 2C). The OPLS-DA model was validated by cross-validation analysis of variance (CV-ANOVA) with a *p*-value of 2.36 × 10^−5^. The results indicated that the S-plot (Figure 2D) of the OPLS-DA pattern and the variable influence on projection (VIP) values were suitable for finding the metabolites responsible for separation [29]. A total of eight metabolites were identified as being significantly different between GF and RF and were signed in the S-plot of the OPLS-DA model (Figure 2D) through independent-sample *t*-test combined with the VIP values (VIP > 1). When GF grew into RF, the levels of cornoside (*p* = 1.14 × 10^−9^), forsythoside A (*p* = 4.16 × 10^−6^), forsythoside C (*p* = 6.55 × 10^−6^), rutin (*p* = 1.33 × 10^−6^), phillyrin (*p* = 1.27 × 10^−4^) and gallic acid (*p* = 5.56 × 10^−4^) decreased significantly, whereas the levels of rengyol (*p* = 3.53 × 10^−8^) and β-glucose (*p* = 1.36 × 10^−5^) increased significantly.

### 2.2. Antioxidant Activity

**Figure 3:**
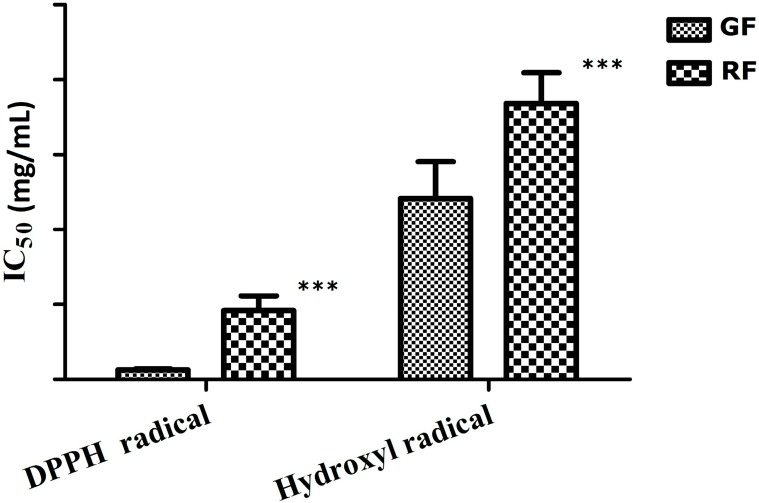
Comparison of antioxidant capacities measured by DPPH and hydroxyl radical scavenging assay between GF and RF. Results are based on independent-sample *t*-test and presented as the mean ± SD (*n* = 10). *** The RF group compared with the GF group, *p* < 0.001.

The antioxidant activity as measured by the ability to scavenge DPPH and hydroxyl radical were compared between 50% methanol extracts of GF and RF. As summarized in Figure 3, GF extracts exhibited lower IC_50_ values for DPPH (0.063 ± 0.007 mg∙mL^−1^) and hydroxyl radical levels (1.207 ± 0.246 mg∙mL^−1^), which indicated that GF showed higher DPPH and hydroxyl radical scavenging activities. In addition, FF contains many phenolic compounds, many of which show high antioxidant properties *in vitro*. Total phenolic content of GF and RF extracts were measured by the Folin-Ciocalteu method (Supplementary Material). The correlation coefficients between antioxidant activity and total phenolic content of GF and RF extracts were calculated. Significant positive correlations were observed between the total phenolic content and 1/IC_50_ values for DPPH (*r* = 0.956, *p* < 0.05) and hydroxyl radical (*r* = 0.917, *p* < 0.05) scavenging capacity, which indicated the significant contribution of phenolic compounds to the antioxidant activities of GF and RF.

### 2.3. Antibacterial Activity

The preliminary antibacterial activity of 50% methanol extracts from all samples was performed by the agar well diffusion method. Results showed that GF and RF were active against four tested bacterial strains. To confirm and quantitatively determine the antibacterial activity, the minimum inhibitory concentration (MIC) was determined for four bacterial strains. As shown in Table 2, the MICs of GF and RF against *Staphylococcus aureus* were more sensitive, with values of 3.592 and 3.903 mg∙mL^−1^, respectively. However, no obvious difference was observed between GF and RF in their MIC values against the four tested bacterial strains (*p* > 0.05).

**Table 2:** Minimum inhibitory concentration (MIC) of GF and RF against four bacterial strains (mean ± SD, *n* = 10).

Groups	Minimum Inhibitory Concentration (MIC, mg∙mL^−1^)			
	*Escherichia coli*	*Pseudomonas aeruginosa*	*Staphylococcus aureus*	*Bacillus subtilis*
GF	11.25 ± 2.63	16.25 ± 6.04	3.59 ± 1.48	8.13 ± 3.02
RF	9.37 ± 3.29	15.00 ± 3.29	3.90 ± 1.69	8.75 ± 3.23
Streptomycin	0.097	0.049	0.39	0.049

## 3. Discussions

GF and RF, which can be collected in a month’s interval in autumn, are fruits of *F. suspense* at different maturity stages. They are the official sources of FF. FF undergoes a complex series of physical and biochemical changes, including shape, color, pathogen and resistance, during the process of fruit ripening. Biochemical changes can certainly lead to variation in biological activities, but GF and RF are not distinguished in current clinical use. The quality of *F. suspensa* 70% ethanol extracts was evaluated by HPLC coupled with a photodiode array detector, and the results suggested that the contents of forsythoside A and phillyrin were all higher in GF than in RF [8]. The 12 major constituents of *F. suspensa* were also determined by HPLC, and the findings showed that forsythoside is the dominant compound in FF. The contents of bioactive compounds, such as forsythoside A, rutin and phillyrin, were also higher in GF than in RF [9]. Furthermore, the amino acid and trace element contents of GF and RF were compared; the amino acid content was higher in GF than in RF, whereas the trace element content of RF was greater than that of GF [30]. However, obtaining the metabolic profiles of chemical constituents with all of the above analytical techniques is arduous. Hence, to comprehensively compare the chemical compositions of GF and RF, ^1^H-NMR-based metabolomics coupled with HPLC and UV spectrophotometry was adopted in the current study, and various metabolites were found. The antioxidant and antibacterial activities of 50% methanol extracts of GF and RF were also evaluated *in vitro*.

### 3.1. Metabolic Profiling of Fruits at Different Maturity Stages of F. suspensa (GF and RF)

Many studies have suggested that phenylethanoid glycosides, lignans and flavonoids are the main bioactive components detected by HPLC, which are responsible for the various biological activities of FF [1,2,7,8]. In the present study, metabolic profiling was performed based on HPLC at 235 nm (Figure S4). A total of 26 characteristic peaks, including six characteristic peaks in the chromatograms of GF and RF, were obtained. PCA score plots showed that GF and RF samples could be clearly separated along PC1 (with PC1 and PC2 accounting for 62.6% of total variance; Figure S5A). The corresponding loading plot of PCA (Figure S5B) showed that the contents of forsythoside A, forsythoside C, rutin and phillyrin were higher in GF than in RF, which was consistent with previous reports in the literature [8,9].

Second, NMR-based metabolomics coupled with multivariate data analysis was applied. Metabolomic profiling suggested that the main differences were observed in aqueous methanol fractions of GF and RF, but no obvious difference existed in chloroform fractions. The most relevant variable contributions to the separation of GF and RF are shown in Table 1 and Figure 2D. What is more, more chemical information, including secondary metabolites, such as cornoside and rengyol, and primary metabolites, such as amino acids, sugars and organic acids, were found by NMR compared with HPLC. Cornoside is also a characteristic ingredient of GF, whereas rengyol is a characteristic component of RF. Therefore, NMR-based metabolomics is better-suited for identifying GF or RF in TCM prescriptions.

### 3.2. In Vitro Antioxidant and Antibacterial Activities of Fruits in Different Maturation Stages of F. suspensa (GF and RF)

In this study, antioxidant and antibacterial activity assays were used to evaluate the biological activities of FF *in vitro*. Phenolic compounds, such as forsythoside A, forsythoside C, phillyrin and rutin, which exhibit free radical scavenging activities, were mostly found in GF. When GF had grown to RF, the contents of forsythoside A, forsythoside C, phillyrin and rutin decreased, which corresponded to a reduction in total phenolic content and antioxidant activity. Forsythoside A, forsythoside C, rutin, phillyrin and gallic acid were all found to be present at significantly different levels in metabolites of GF compared with those of RF. However, comparison of their relative contents showed that forsythoside A content was higher than those of other phenolic compounds in GF, which was consistent with previous reports [8,9]. Thus, the higher antioxidant activity of GF compared to that of RF may be attributed to the greater contents of forsythoside A in GF than in RF. The high DPPH and hydroxyl radical scavenging activities of GF suggest that antioxidant drugs can be sourced from GF.

FF also exhibits antibacterial activities in phenylethanoid glycosides, specifically forsythoside A, which is mainly responsible for the antibacterial activities of GF [31]. However, in the current study, no significant difference was observed in the antibacterial activities of GF and RF, although GF was found to have a higher level of forsythoside A than RF. Thus, other RF compounds responsible for its antibacterial activity should be further investigated. In addition, neither GF nor RF can affect fungi. To determine the differences in biological activities between GF and RF more effectively, some *in vivo* pharmacological models that conform to FF against the main TCM clinical symptoms should be used in future research.

### 3.3. Putative Biosynthetic Pathways of the Major Secondary Metabolites in F. suspensa

**Figure 4:**
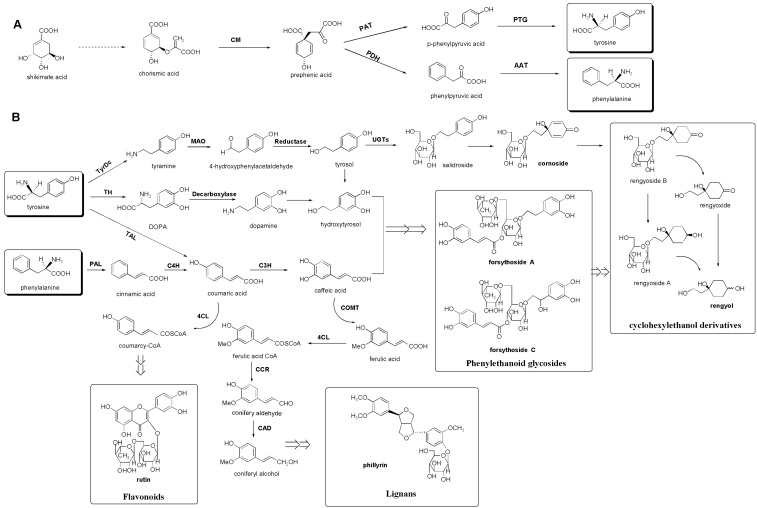
Putative biosynthetic pathways of the major secondary metabolites in FF. The shikimate pathway provides a route to tyrosine and phenylalanine (**A**); Tyrosine and phenylalanine are starting points in the biosynthesis of several major secondary metabolites in FF (**B**). Enzyme names are abbreviated as follows: CM, chorismate mutase; PDH, prephenate dehydratase; PAT, prephenate aminotransferase; AAT, aromatic aminotransferase; PTG, pretyrosine dehydrogenase; TyrDC, tyrosine decarboxylase; MAO, monoamine oxidase; UGTs, UDP-glycosyltransferases; TH, tyrosine hydroxylase; TAL, tyrosine ammonia lyase; PAL, phenylalanine ammonia lyase; C4H, pretycinnamate-4-hydroxylase; 4CL, 4-coumarate: coenzyme A ligase; C3H, coumarate 3-hydroxylase; COMT, caffeic acid O-methyltransferase; CCR, cinnamoyl-CoA reductase; CAD, cinnamyl alcohol dehydrogenase.

The metabolic profiles of FF performed in this study using NMR-based metabolomics coupled with HPLC showed that phenylethanoid glycosides, lignans, cyclohexylethanol derivatives and flavonoids are the major secondary metabolites in FF. These findings are consistent with those of previous studies on the chemical composition of FF. The biosynthesis of these above kinds of compounds begins with the generation of phenylalanine and tyrosine precursors via the shikimic acid pathway (Figure 4) [32,33]. The biosynthesis pathways of lignans and flavonoids have been systematically studied, and the enzymes involved, such as pretycinnamate-4-hydroxylase (C4H), 4-coumarate: coenzyme A ligase (4CL), coumarate 3-hydroxylase (C3H) and caffeic acid *O*-methyltransferase, have been confirmed [32,34,35]. However, the biosynthetic pathways of phenylethanoid glycosides and cyclohexylethanol derivatives have not been studied thoroughly, and the enzymatic steps remain unclear. The caffeoyl moiety of phenylethanoid glycosides might be synthesized from phenylalanine via a cinnamic acid pathway. The hydrotyrosol moiety of phenylethanoid glycosides might be derived from tyrosine, either through tyramine or dopamine, based on the biosynthesis of phenylethanoid glycosides in other plants, such as verbascoside and echinacoside [36,37]. Hu *et al*. reported that tyrosine is a better precursor than phenylalanine for phenylethanoid glycoside accumulation and that the rate-limiting enzymes may be in the tyrosine branches [38]. The biosynthetic pathways of cyclohexylethanol derivatives may start from tyrosol and lead via salidroside to cornoside before further transforming to rengyol [25]. Endo *et al*. [26,39,40] have elucidated the biogenetic transformations from salidroside via cornoside to rengyol and the related natural cyclohexylethanoids by oxidation and successive reduction. However, all of the above findings are speculative, and real biosynthetic details remain unknown.

Our results showed that GF contained high levels of secondary metabolites, such as forsythoside A, cornoside, rutin and phillyrin, whereas RF contained a high level of rengyol (Table 1). This suggests that the enzymes catalyzing conversion of cornoside to rengyol may be upregulated during ripening. Phenylethanoid glycosides, such as forsythoside A and forsythoside C, may also be significantly decomposed by the enzyme and degraded finally into rengyol. Moreover, the contents of phillyrin and rutin, which have been shown to mediate various biological activities of FF [7], were decreased from GF to RF stages. Hence, cornoside may be accumulated in the early mature stage of the fruit (GF), whereas cyclohexylethanol derivatives, such as rengyol, may be accumulated in the completely mature stage of the fruit (RF).

RF is also commonly used in Japan, where FF is referred to as ‘rengyo’ and RF contains higher levels of rengyol than GF [33,39]. Thus, some contradictions remain in the exact clinical usage of FF, and further studies are needed for clarification on this issue.

## 4. Experimental Section

### 4.1. Chemicals and Reagents

Caffeic acid, rutin and phillyrin were purchased from the National Institute for the Control of Pharmaceutical and Biological Products (NICPP, Beijing, China). Forsythoside A, forsythoside C and adoxosidic acid were isolated from FF in our laboratory; their structures were elucidated by direct comparison of their UV, IR, NMR and MS spectral data with those in the literature [18,19,22]. HPLC analysis showed that their purities were over 95%. HPLC-grade methanol was obtained from Fisher Scientific (Fisher Scientific, Fair Lawn, NJ, USA). Deuterated chloroform (CDCl_3_, 99.8% D) containing tetramethylsilane (TMS, 0.03%, *m*/*v*) and methanol-d_4_ (99.8% D) were obtained from Merck (Darmstadt, Germany). D_2_O was bought from Norell (Landisville, NJ, USA). Sodium 3-trimethlysilyl [2,2,3,3,] propionate (TSP) was obtained from Cambridge Isotope Laboratories, Inc. (Andover, MA, USA). NaOD was purchased from Armar (Dottingen, Switzerland), and 2,2-diphenyl-1-picrylhydrazyl (DPPH), H_2_O_2_ (30%), 2-deoxyribose, Folin-Ciocalteu reagent and gallic acid were purchased from Sigma Chemical Co. (St. Louis, MO, USA). All other solvents and chemicals were of analytical grade. All spectrophotometric data were acquired using a Cary 50 scan UV-VIS spectrometer (Varian Associates Inc., Palo Alto, CA, USA).

### 4.2. Plant Materials

Up to 20 commercial FF samples (Table 3) consisting of 10 GF and 10 RF were collected from 10 different counties located in Southeast Shanxi Province of China, which are considered the main producing areas of FF. The samples were authenticated by Prof. Xue-Mei Qin of Shanxi University. All voucher specimens were deposited in the herbarium in the Institute of Molecular Science of Shanxi University, China. All samples were ground to a fine powder and sieved with a bolt (20 meshes). The powder was then kept in an airtight container at −80 °C until use.

**Table 3:** Representative samples of GF and RF in this study.

No.	Habitat	Collection Time	Growing Pattern
GF01	Anze, Shanxi, China	5 September 2013	wild
GF02	Guxian, Shanxi, China	10 September 2013	wild
GF03	Fushan, Shanxi, China	7 September 2013	wild
GF04	Lingchuan, Shanxi, China	9 September 2013	wild
GF05	Pingshun, Shanxi, China	11 September 2013	wild
GF06	Qinshui, Shanxi, China	6 September 2013	wild
GF07	Zhangzi, Shanxi, China	September 13, 2013	wild
GF08	Tunliu, Shanxi, China	13 September 2013	wild
GF09	Zuoquan, Shanxi, China	12 September 2013	wild
GF10	Licheng, Shanxi, China	12 September 2013	wild
RF01	Anze, Shanxi, China	9 October 2013	wild
RF02	Guxian, Shanxi, China	9 October 2013	wild
RF03	Fushan, Shanxi, China	10 October 2013	wild
RF04	Lingchuan, Shanxi, China	11 October 2013	wild
RF05	Pingshun, Shanxi, China	17 October 2013	wild
RF06	Qinshui, Shanxi, China	17 October 2013	wild
RF07	Zhangzi, Shanxi, China	15 October 2013	wild
RF08	Tunliu, Shanxi, China	21 October 2013	wild
RF09	Zuoquan, Shanxi, China	20 October 2013	wild
RF10	Licheng, Shanxi, China	20 October 2013	wild

4.3. ^1^H-NMR Data Acquisition and Analysis

Two different extraction procedures were used for NMR analysis [21]. All powder samples (200 mg each) were transferred into 10-mL glass centrifuge tubes. About 6 mL of CHCl_3_–MeOH–H_2_O (2:1:1, v/v/v) mixture was added to the tube, followed by vortexing for 1 min and ultrasonication for 30 min. The material was then centrifuged at 3500 rpm for 25 min. Chloroform and aqueous methanol fractions were transferred separately into a 25-mL round-bottomed flask and dried using a rotary vacuum evaporator. Chloroform fractions were dissolved in 800 µL CDCl_3_, and aqueous methanol fractions were dissolved in 800 µL mixture (1:1) of CD_3_OD and KH_2_PO_4_ buffer in D_2_O (adjusted to pH 6.0 by 1 N NaOD) containing 0.05% TSP. The samples were then centrifuged for 10 min at 13,000 rpm, and supernatants (600 μL) were transferred into 5-mm tubes for NMR analysis.

^1^H-NMR was recorded at 25 °C on a Bruker 600MHz AVANCE III NMR spectrometer (600.13 M proton frequencies). CD_3_OD and CDCl_3_ were used for internal lock purposes. Each ^1^H-NMR spectrum consisted of 64 scans, which required 5 min of acquisition time with the following parameters: 0.18 Hz/point, pulse width = 30° (12.7 μs) and relaxation delay = 5.0 s. A presaturation sequence was used to suppress the residual H_2_O signal with low-power selective irradiation at H_2_O frequency during the recycle delay. Free induction decays (FIDs) were Fourier transformed with Luria–Bertani (LB) = 0.3. A mixture of CHCl_3_–MeOH–H_2_O (2:1:1) was selected as the extraction system for FF. The resulting spectra were manually phased, baseline-corrected and calibrated to TSP at 0.00 ppm for aqueous methanol fractions and TMS at 0.00 ppm for chloroform fractions.

The ^1^H-NMR spectra were processed using MestReNova software (Version 8.0.1, Mestrelab Research, Santiago de Compostela, Spain). For aqueous methanol fractions, spectral intensities were scaled to TSP and reduced to integrated regions of equal width (0.04 ppm) corresponding to the region of δ 0.50–10.02. The regions of δ 4.70–5.02 and δ 3.30–3.38 were excluded from the analysis because of residual signals of D_2_O and CD_3_OD, respectively. For the chloroform fractions, spectral intensities were scaled to TMS and reduced to integrated regions at 0.04 ppm corresponding to the region of δ 10.02–0.50. The region between δ 7.22 and δ 7.30 was removed from the analysis, because of the residual signal of CHCl_3_. The ^1^H-NMR data obtained after mean-centering and Pareto scaling were imported into SIMCA-P 13.0 (Umetrics, Umeå, Sweden), which was then used for multivariate statistical analyses, including PCA, PLS-DA and OPLS-DA. The corresponding variables with variable importance on projection (VIP) values were calculated in the OPLS-DA model. The OPLS-DA, S-plot and VIP value were used for selecting the metabolites responsible for sample differentiation. Metabolites were chosen as differentiating metabolites when their VIP values were larger than 1.0 [41,42]. Statistical analysis was also performed using an independent-sample *t*-test (SPSS 16.0, SPSS Inc., Chicago, IL, USA). In comparisons of differences between two groups, *p* < 0.05 was considered statistically significant.

### 4.4. Antioxidant Activity

All powders (0.10 g each) were accurately weighed and mixed with 10 mL of 50% methanol in centrifuge tubes and ultrasonically extracted (KQ-250DB ultrasonic bath, Kunshan, Jiangsu, China) for 30 min. The resultant mixture was adjusted to the original weight and centrifuged at 3000 rpm for 10 min. The collected supernatant was used as a stock solution to determine antioxidant activity.

#### 4.4.1. DPPH Radical Scavenging Assay

The antioxidant activity of the above extract was determined by the DPPH radical scavenging assay as described before with slight modifications [43,44]. The stock solution (10 mg∙mL^−1^) was diluted with methanol to final concentrations ranging from 0.010 mg∙mL^−1^ to 0.50 mg∙mL^−1^. Methanol was used as a negative control, while ascorbic acid served as a positive control. Half inhibitory concentration (IC_50_) values were also calculated.

#### 4.4.2. Hydroxyl Radical Scavenging Assay

Hydroxyl radical scavenging activity was evaluated by the 2-deoxyribose oxidative degradation assay [45]. The reaction system contained 2.9 mL of KH_2_PO_4_–K_2_HPO_4_ buffer (10.0 mM, pH 7.2), 150 μL of Fe^2+^ (2 mM), 1.0 mL of samples at different concentrations (1.0 mg∙mL^−1^ to 10 mg∙mL^−1^), 150 μL of 2-deoxyribose (200 mM) and 150 μL of ascorbic acid (40 mM). The reaction was initiated by adding 150 μL of H_2_O_2_(10 mM), allowed to run for 10 min at 37 °C and stopped by adding 250 μL of 10% (*w*/*v*) trichloroacetic acid, followed by 250 μL of 1% 2-thiobarbituric acid (*w*/*v*, in 40 mM NaOH). After heating at 80 °C for 20 min followed by rapid ice-cold wash, absorbance at 532 nm was measured against a blank (comprised of the same solution without reagents). All of the reagents were freshly prepared, and IC_50_ values were calculated.

### 4.5. Antibacterial Activity

All powders (2.0 g each) were accurately weighed and extracted with 50% methanol (1:10 *w*/*v*) in a 50-mL round-bottom flask by refluxing for 1 h. The extract solvents were evaporated under reduced pressure using a rotary vacuum evaporator at 50 °C. Residues were dissolved in sterile water at 200 mg∙mL^−1^ of dried herbs, then sterilized before use.

The minimum inhibitory concentration (MIC) was determined by the microdilution bioassay in 96-well microplates [44,46,47] with some modifications. Briefly, two-fold serial dilutions of each extract were added to LB broth to yield volumes of 100 μL, with final concentrations ranging from 0.20 mg∙mL^−1^ to 100 mg∙mL^−1^, and about 100 μL of bacterial culture (approximately 10^5^ CFU/mL) were added into the microplate wells. The microplates were covered and incubated overnight at 37 °C. About 40 μL of 0.2 mg∙mL^−1^*p*-iodonitrotetrazolium chloride were added to the microplate wells and incubated at 37 °C for 30 min to induce bacterial growth. The bacterial suspension of the microplate wells where bacterial growth occurred changed to red. Streptomycin (100 mg∙mL^−1^) was used as a positive control for each bacterium, while solvent and bacterium-free wells served as negative controls. MIC was calculated as the lowest concentration that completely inhibited bacterial growth. Samples were tested against two Gram-negative bacteria (*Escherichia coli* and *Pseudomonas aeruginosa*) and two Gram-positive bacteria (*Staphylococcus aureus* and *Bacillus subtilis*), all of which were obtained from Jun Xu of the College of Life Science, Shanxi University, China. All experiments were performed in triplicate.

### 4.6. Data Analysis

All data are presented as means (mean ± SD) of three measurements. The IC_50_ values were calculated using Origin 8.0 (Microcal Software, Inc., Northampton, MA, USA) by plotting percentage inhibition against concentration. The correlation values were examined using Pearson correlation. All statistical analysis of the data was performed by the independent-sample *t*-test, and Pearson’s correlation coefficient (*r*) was calculated using SPSS 16.0 (SPSS Inc., Chicago, IL, USA). Differences at *p* < 0.05 were considered significant.

## 5. Conclusions

In this study, we first conducted aqueous methanol metabolic profiling using NMR for the analysis of *F. suspensa* fruits. *In vitro* biological activities, such as antioxidant and antibacterial effects, were then measured. Total phenolic content in GF and RF were also determined. A total of 27 metabolites were identified from our NMR data, and eight of them were different between the GF and RF groups. The GF group contained a higher level of forsythoside A, forsythoside C, cornoside, rutin, phillyrin and gallic acid, as well as a lower level of rengyol and β-glucose compared to the RF group. In addition, antioxidant activity was much higher in GF, and no significant difference was found between the antibacterial activities of GF and RF.

Despite our knowledge of chemical differences between GF and RF, no distinctions in their clinical efficacy have been established to date. In order to guarantee the efficacy and safety of FF in clinical applications, a systematic investigation of differences in the chemical and biological activities of GF and RF is warranted. Furthermore, since FF is used commonly in TCM, further study on the difference in effectiveness of GF and RF in TCM prescriptions is also necessary.

## Supplementary Materials

Supplementary materials can be accessed at: http://www.mdpi.com/1420-3049/20/06/10065/s1.
