# Supplementary material for: Comparison of Fruits of Forsythia suspensa at Two Different Maturation Stages by NMR-Based Metabolomics
Source: Molecules. 2015 May 29;20(6):10065–81. doi: 10.3390/molecules200610065 (PMC6272181; doi:10.3390/molecules200610065)
Supplement: Supplementary file 1 [file molecules-20-10065-s001.pdf]

Supplementary Material of

Comparison of Fruits of *Forsythia suspensa* at Two Different Maturation Stages by NMR-Based Metabolomics

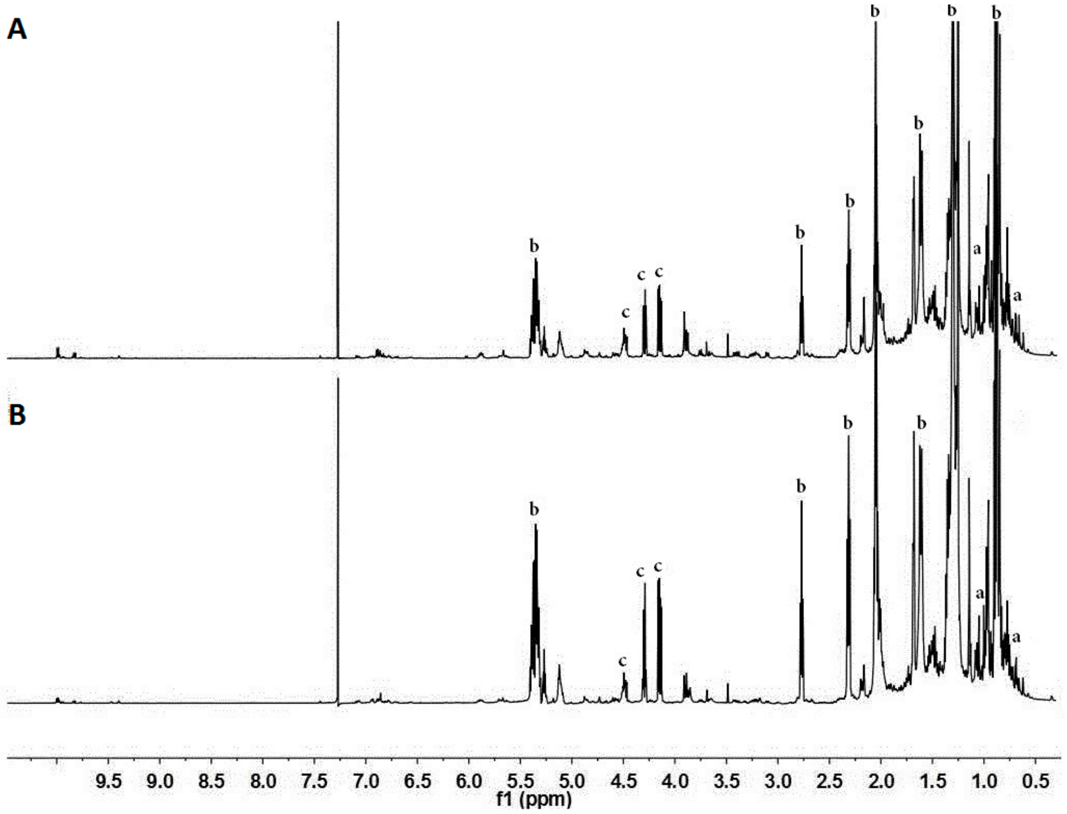

Figure S1. Representative total ^{1}H-NMR spectra of chloroform fractions of ripe Fructus Forsythiae (**A**) and green Fructus Forsythiae (**B**). a, β-sitosterol; b, fatty acids; c, glycerol esters.

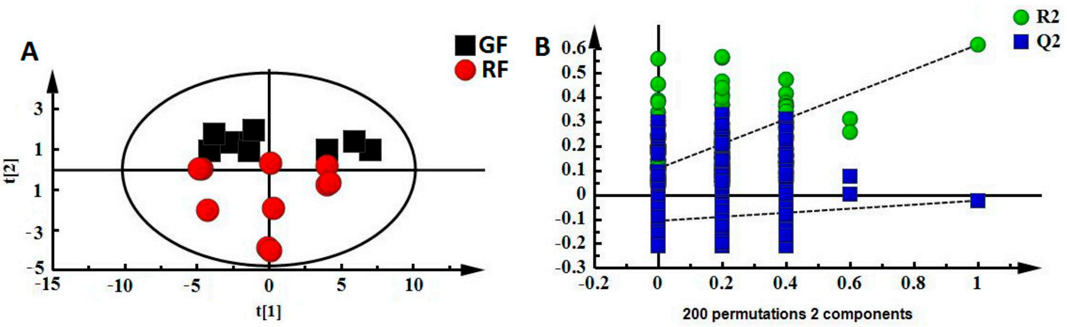

Figure S2. Score plot (**A**) of principal component analysis (PCA) and permutation test (**B**) results obtained from ^{1}H-NMR spectra of the chloroform fraction of green Fructus Forsythiae (GF) and ripe Fructus Forsythiae (RF): GF, black square; RF, red dot.

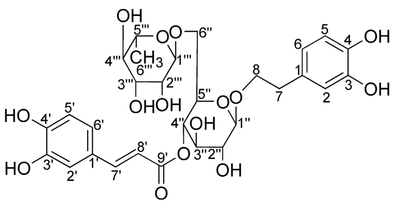

Forsythoside A

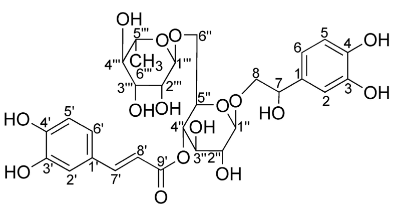

Forsythoside C

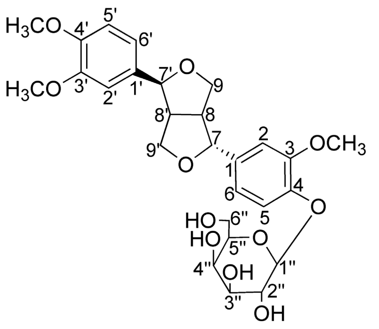

Phillyrin

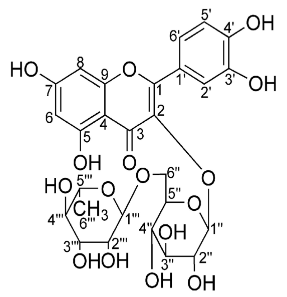

Rutin

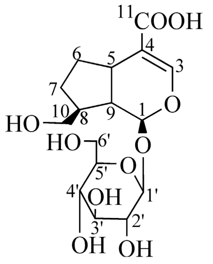

Adoxosidic acid

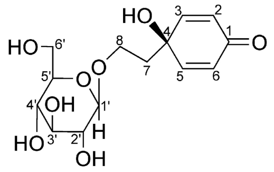

Cornoside

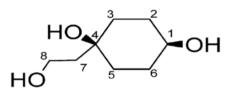

Rengyol

Figure S3. Chemical structures of secondary metabolites identified by ^{1}H-NMR spectra in FF extracts.

1. HPLC Analysis

1.1. Sample Solution Preparations

All ground samples (0.10 g each) were accurately weighed and extracted by refluxing with 20 mL 50% methanol solution for 1 h. The resultant mixture was adjusted to the original weight, and aliquots of the supernatant were filtered through a 0.22-μm membrane before use; about 10 μL were injected into the HPLC instrument for analysis.

1.2. Chromatographic System

The HPLC method, which was validated by defining the linearity, limits of quantification and detection, repeatability, precision, stability and recovery was based on a previous description [1,2] with some modifications. A Waters Alliance e2695 instrument equipped with a 2998 photodiode array detector was used for analyses. System control and data analysis were carried out using Empower 3 software (Waters, Milford, MA, USA). The separation was carried out on a Venusil MP C18 column (100 mm × 2.1 mm; i.d., 3 μm; Agela, Wilmington, DE, USA). The mobile phase consisted of 0.3% acetic acid in water (A) and methanol (B). Gradient elution was set as follows: 10%–20% B in 0–3 min,

20%–30% B in 3–5 min, 30%–33% B in 5–8 min, 33%–37% B in 8–15 min, 37%–40% B in 15–20 min, 40%–52% B in 20–25 min, 52%–64% B 25–30 min and 64%–71% B in 30–35 min; and then balanced for 5 min. The mobile phase flow rate was set at 0.3 mL·min^{-1}. The photodiode array (PDA) detector was set at 235 nm, and the on-line UV spectra were recorded in the range of 195–400 nm. The column temperature was kept at 30 °C.

1.3. Data Analysis

HPLC chromatographic data were analyzed using professional software, Similarity Evaluation System for Chromatographic Fingerprint of TCM (Version 2008), which was recommended by the Chinese Pharmacopoeia Committee and the State Food and Drug Administration of China. The respective retention times and peak areas for HPLC were obtained. The similarity between different chromatograms was compared with the mean chromatograms among tested samples. The peak areas were normalized and then used as input data for PCA analysis. PCA with Pareto scaling was performed with SIMCA-P13.0 (Umetrics, Umeå, Sweden).

1.4. Metabolic Profiling by HPLC

Several studies have suggested that phenylethanoid glycosides, lignans and flavonoids are the main bioactive components that are responsible for various biological activities of FF [3–5], which can be detected by HPLC. A total of 26 characteristic peaks were found in the chromatograms of GF and RF, of which six characteristic peaks were assigned as caffeic acid (Peak 10), forsythoside C (Peak 15), forsythoside A (Peak 19), rutin (Peak 21), phillyrin (Peak 23) and adoxosidic acid (Peak 26), by comparing the UV spectra and their retention time with those of the reference compounds (Figure S4). Forsythoside A (Peak 19), an important active constituent in FF, was the most dominant compound in all samples; thus, it was used to calculate the relative retention time, which was a useful index to distinguish the peaks with minor errors in retention time. Both GF and RF showed consistent and high similarity (GF, approximately 0.95; RF, approximately 0.90). Visual inspection indicated that most of the peak areas of detected compounds were larger in GF than RF, except for Peaks 7, 13 and 17. PCA, a non-parametric method of classification and a sophisticated technique that is widely used in reducing the dimensions of multivariate problems, showed that GF and RF samples can be separated clearly on PC1 (with PC1 and PC2 accounting for 62.6% of the total variance). The corresponding loading plot (Figure S5B) indicated that the GF contained more forsythoside A and forsythoside C Peaks 6, 9, 14, 16 and 18 than RF, whereas RF contained more Peaks 7, 13, and 17 than GF.

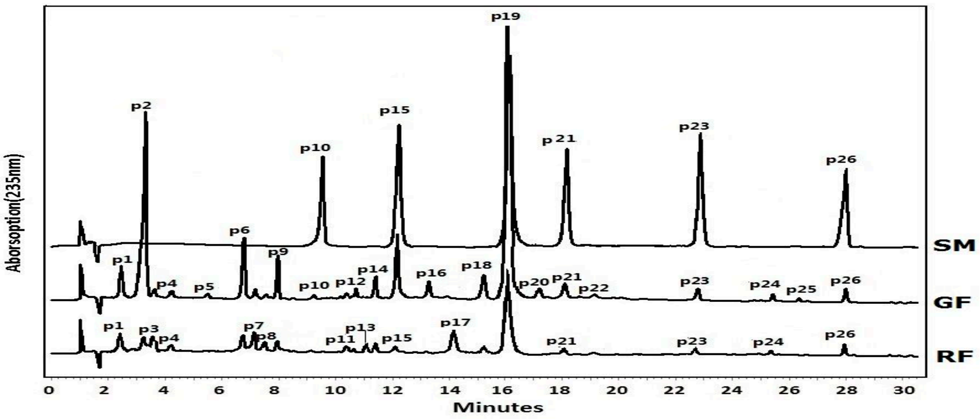

Figure S4. HPLC chromatograms (UV chromatogram at 235 nm) of GF, RF and standard mixture (SM): caffeic acid (Peak 10), forsythoside C (Peak 15), forsythoside A (Peak 19), rutin (Peak 21), phillyrin (Peak 23) and adoxosidic acid (Peak 26).

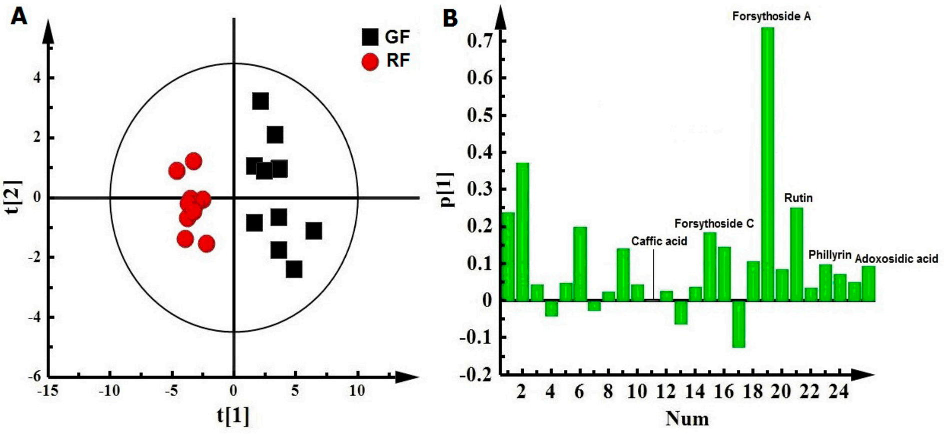

Figure S5. Score plot (**A**) and loading plot (**B**) of principal component analysis (PCA) results obtained from HPLC data derived from 50% methanol extracts of green Fructus Forsythiae (GF) and ripe Fructus Forsythiae (RF): GF, black square; RF, red dot.

2. Determination of Total Phenolic Content

2.1. Sample Solution Preparations

All samples (0.10 g each) were accurately weighed and mixed with 10 mL of 50% methanol in a centrifuge tube and ultrasonically extracted (KQ-250DB ultrasonic bath, Kunshan, Jiangsu, China) for 30 min, respectively. The resultant mixture was adjusted to the original weight and centrifuged at 3000 rpm for 10 min. The collected supernatant as the stock solution was used to determine the total phenolic content.

2.1. Folin-Ciocalteu Method

Total phenolic content of the sample was determined according to as previously described with some modifications [6]. Gallic acid was used as a standard for plotting the calibration curve ($y = 0.031x + 0.060$, $r = 0.9991$). The total phenolic contents were calculated on the basis of the gallic acid calibration curve. The results were expressed in mg of gallic acid equivalent per gram of dried plant material (Table S1).

Table S1. Total phenolic content of GF and RF ($n = 10$).

Groups	Total Phenolic Content ($mg \cdot g^{-1}$)	Antioxidant Activity (IC_{50}, $mg \cdot mL^{-1}$)	
		DPPH Radical	Hydroxyl Radical
GF	80.829 ± 10.110	0.063 ± 0.007	1.207 ± 0.246
RF	23.536 ± 7.988 **	0.460 ± 0.098 **	1.841 ± 0.206 **

** RF group compared with GF group, $p < 0.01$.

References

1. Xia, Y.G.; Yang, B.Y.; Wang, Q.H.; Liang, J.; Wei, Y.H.; Yu, H.D., Zhang, Q.B.; Kuang, H.X. Quantitative analysis and chromatographic fingerprinting for the quality evaluation of *Forsythia suspensa* extract by HPLC coupled with photodiode array detector. *J. Sep. Sci.* **2009**, *32*, 4113–4125.
2. Guo, H.; Liu, A.H.; Li, L.; Guo, D.A. Simultaneous determination of 12 major constituents in Forsythia suspensa by high performance liquid chromatography-DAD method. *J. Pharm. Biomed. Anal.* **2007**, *43*, 1000–1006.
3. Nishibe S.; Okabe, K.; Tsukamoto, H.; Sakushima, A.; Hisada,S.; Baba, H.; Akisada, T. Studies on the Chinese crude drug Forsythiae Fructus VI. The structure and antibacterial activity of suspersaside isolated from Forsythia Suspensa. *Chem. Pharm. Bull.* **1982**, *30*, 4548–4553.
4. Ozaki,Y.; Rui, J.; Tang, Y.; Satake, M. Antiinflammatory effect of Forsythia suspensa Vahl and its active fraction. *Biol. Pharm. Bull.* **1997**, *20*, 861–864.
5. Rahman, M. M. A., Dewick, P. M., Jackson, D. E., Lucas, J.A. Production of lignans in *Forsythia intermedia* cell cultures. *Phytochemistry* **1990**, *29*, 1861–1866.
6. Wang, Y.; Wang, R.B.; Sun, L.; Wang, B.H.; Ding, Y.X.; Xu, L.Q; Tian, S. Relationship between total flavonoids，total phenolics and 2, 2-diphenyl-1-picrylhydrazyl radical scavenging activities during growth of Forsythia suspensa leaves. *Chin. J. Exp. Tradit. Med. Form.* **2011**, *8*, 109–112. (In Chinese)
